# Genomic Evolution of *Saccharomyces cerevisiae* under Chinese Rice Wine Fermentation

**DOI:** 10.1093/gbe/evu201

**Published:** 2014-09-10

**Authors:** Yudong Li, Weiping Zhang, Daoqiong Zheng, Zhan Zhou, Wenwen Yu, Lei Zhang, Lifang Feng, Xinle Liang, Wenjun Guan, Jingwen Zhou, Jian Chen, Zhenguo Lin

**Affiliations:** ^1^Department of Bioengineering, School of Food Sciences and Biotechnology, Zhejiang Gongshang University, Hangzhou, China; ^2^Key Laboratory of Industrial Biotechnology, School of Biotechnology, Jiangnan University, Wuxi, China; ^3^College of Life Sciences, Zhejiang University, Hangzhou, China; ^4^Department of Biology, Saint Louis University; ^5^Department of Ecology and Evolutionary Biology, Rice University

**Keywords:** genome, evolution, RNA-Seq, rice wine, *Saccharomyces cerevisiae*

## Abstract

Rice wine fermentation represents a unique environment for the evolution of the budding yeast, *Saccharomyces cerevisiae*. To understand how the selection pressure shaped the yeast genome and gene regulation, we determined the genome sequence and transcriptome of a *S. cerevisiae* strain YHJ7 isolated from Chinese rice wine (Huangjiu), a popular traditional alcoholic beverage in China. By comparing the genome of YHJ7 to the lab strain S288c, a Japanese sake strain K7, and a Chinese industrial bioethanol strain YJSH1, we identified many genomic sequence and structural variations in YHJ7, which are mainly located in subtelomeric regions, suggesting that these regions play an important role in genomic evolution between strains. In addition, our comparative transcriptome analysis between YHJ7 and S288c revealed a set of differentially expressed genes, including those involved in glucose transport (e.g., *HXT*2, *HXT7*) and oxidoredutase activity (e.g., *AAD*10, *ADH*7). Interestingly, many of these genomic and transcriptional variations are directly or indirectly associated with the adaptation of YHJ7 strain to its specific niches. Our molecular evolution analysis suggested that Japanese sake strains (K7/UC5) were derived from Chinese rice wine strains (YHJ7) at least approximately 2,300 years ago, providing the first molecular evidence elucidating the origin of Japanese sake strains. Our results depict interesting insights regarding the evolution of yeast during rice wine fermentation, and provided a valuable resource for genetic engineering to improve industrial wine-making strains.

## Introduction

As one of the oldest alcoholic beverages in human history, the Chinese rice wine (Huangjiu) has been brewed and consumed for more than 5,000 years (McGovern et al. 2004). Huangjiu is typically fermented from rice with wheat *Qu* (*koji* in sake) and the budding yeast *Saccharomyces cerevisiae*, which is the most dominant microorganism in rice wine fermentation processes. The wheat *Qu* contains many molds (fungi), such as *Aspergillus oryzae*, which break down starches to sugars and digest proteins to peptides or amino acids. The sugars are further fermented by yeasts to produce alcohols. The combination of progressive saccharification of starches and alcoholic fermentation is called “parallel fermentation” (supplementary fig. S1, Supplementary Material online). The parallel fermentation process avoids exposure of yeast cells to high glucose content and results in high ethanol production, which can be >20% (v/v) in the final fermentation must (Chen and Xu 2010; Akao et al. 2011). Moreover, the formation of sensory characteristics of Chinese rice wine is influenced by different *S*. *cerevisiae* strains that produce different flavor compounds, such as higher alcohols, acetates, ethyl esters, and aldehydes (Chen and Xu 2010). To control the quality of wine product, different *S*. *cerevisiae* strains have been selected as starter cultures by the wine-maker to manipulate the influence of yeasts.

Huangjiu has been regarded as a alcohol beverage with high nutritional and pharmacological values (Xie 2008). However, fermentation of Huangjiu may also produce some undesired byproducts, including higher alcohols and ethyl carbamate (Zhao et al. 2013). Higher alcohols (e.g., isoamyl alcohol, phenylethyl alcohol) may trigger a headache in people after consumption of Huangjiu, whereas ethyl carbamate is possibly carcinogenic to humans. A better understanding of genetic basis responsible for the metabolism of these undesired byproducts is necessary to reduce their production. Furthermore, as industrial *S. cerevisiae* strains have been adapted to the specific wine brewing environmental conditions, their genomes might have been subjected to strong selective pressures (Querol et al. 2003). A complete sequenced genome of Huangjiu strain may provide a better understanding of the genetic basis of the strain for adaptation to specific fermentation environments. However, although the genomes of many *S. cerevisiae* strains have been completely sequenced, including a Japanese sake strain K7 (Akao et al. 2011; Borneman et al. 2011, 2012; Babrzadeh et al. 2012; Nijkamp et al. 2012; Brown et al. 2013; Treu et al. 2014), the genome sequences of Huangjiu strain have not yet been determined. In addition, although the brewing processes of Japanese sake and Huangjiu are similar (supplementary fig. S1, Supplementary Material online), their sensory characteristics and nutrients are quite different, and the genetic basis leading to such differences remains unexamined. Comparative studies of genomes and transcriptomes are indispensable to unravel the underlying genetic variations responsible for the unique sensory characteristics and nutrients of Huangjiu, which will pave the way for genetic manipulation of yeast strains to improve their product quality.

In this study, we sequenced the genome and transcriptome of a Huangjiu strain YHJ7, and compared it with the laboratory strain S288c, Japanese sake strain K7, and a Chinese industrial bioethanol strain YJSH1. We identified many single nucleotide polymorphisms (SNPs)/InDels, gene loss and gains, and differentially expressed genes. In addition, many of these genomic variations are likely associated with the adaption to Huangjiu fermentation environment. Furthermore, our molecular phylogeny analysis suggested that the Japanese sake strains might have originated from Huangjiu strains about 2,300 years ago, which is consistent with the historical record about the ancient cultural interactions between the two countries.

## Materials and Methods

### Strains and Growth Conditions

The haploid strain *S. cerevisiae* YHJ7 was generated by sporulation from a strain isolated from Huangjiu fermentation sample (Li et al. 2013). Strains were routinely grown in YPD medium (1% yeast extract, 1% peptone, and 1% glucose) at 28 °C and shaken at 200 rpm. The genomic DNA of YHJ7 strain was extracted from cells in midexponential phase (∼18 h), using yeast DNA extraction kits as per manufacturer’s instructions (Tiandz, Beijing), and used for Illumina sequencing.

### Genome Sequencing and Reads Preprocessing

Library construction was followed by Illumina sample preparation kit instructions, and libraries were sequenced on the Illumina Hiseq 2000 at Beijing Genome Institute. The raw reads from both DNA and RNA were first assessed for their quality using FastQC (http://www.bioinformatics.babraham.ac.uk/projects/fastqc/, last accessed September 17, 2014), and showed base bias in the first few bases of the reads and poor quality in the last few bases. Bad quality reads (phred score < 20) were filtered, and the first and last few bases of reads were trimmed using PRINSEQ (Schmieder and Edwards 2011), if those reads cannot be mapped to S288c reference genome. This Whole Genome Shotgun project has been deposited at DDBJ/EMBL/GenBank under the BioProject accession number PRJNA169002.

### SNP and InDel Calling

Short reads were mapped to the genome of the reference strain S288c (obtained from the Saccharomyces Genome Database, June 2013), using the mapping tools BWA (version 0.52) and Bowtie2. SNP and InDel calls were made using the “mpileup” options of SAMtools version 0.1.8 (Li et al. 2009), followed by bcftools and the vcfutils.pl script with “varFilter” options (vcftools.sourceforge.net). SNPs were called only for positions with a minimal mapping quality (-Q) of 20, and maximum read depth (-D) was set at 200. The identified SNPs or InDels in both mapping methods were checked for their overlap using intersectBed of BEDtools (Quinlan and Hall 2010). The SNPs and InDels were classified as coding region and intergenic region, according to their positions in the reference S288c genome. SNPs in the coding sequences were further annotated as synonymous or nonsynonymous substitutions using ANNOVAR package (Wang et al. 2010). Gene Ontology (GO) term enrichment analysis was carried out by the GO terms finder in SGD website (http://www.yeastgenome.org/cgi-bin/GO/goTermFinder.pl, last accessed September 17, 2014).

For detection of larger InDels, Delly (v0.011) (Rausch et al. 2012) and Pindel (v0.2.2) (Ye et al. 2009) were used to call variants from the paired-end reads with default parameters (median size of 350). Deletions and insertions of at least 100 bp in size and detected by both methods were considered for further analysis. To prevent false positives, genes located at these deletions with support of more than five reads and with alignment quality higher than 20 were considered.

### Genome Assembly and Annotation

De novo assemblies were performed with Velvet (version 1.2.09) (Zerbino and Birney 2008). After running several assemblies, we found that a K-mer size of 65 was giving the best results in terms of *N*_50_ contig and total assembly length after scaffolding and gap filling. Reference-guided assemblies were carried out by the CLC Genomics Workbench v. 6.05 (CLC BIO, Aarbus, Denmark). The combination of de novo assembly and reference-guided assembly was performed manually by using microbial genome finishing module in CLC genomics workbench. As this strain was closely related to another Chinese strain YJSH1 (Zheng et al. 2012) (sequenced in our laboratory before), we first used the genome of YJSH1 as reference genome, then some contigs that cannot be aligned to YJSH1 consensus sequence were aligned to S288c consensus.

Genome annotation was based on a combination of ab initio prediction (minimum size, 150 bp) and comparative gene prediction by direct mapping of S288c open reading frames (ORFs) from the Saccharomyces Genome Database. Initial ORFs were predicted using the AUGUSTUS (Stanke et al. 2004) and Glimmer (Delcher et al. 1999) with the annotated ORFs of S288c being used to build the prediction model. The final ORFs were selected manually by combining the ab initio and comparative prediction methods. ORF names were assigned with their closest S288c homolog. The ORFs with no match to S288c were searched against the nonredundant protein database to identify a closest existing homology match. The complete ORF annotations were available in supplementary data set S1, Supplementary Material online.

### RNA Sequencing Analysis

RNA sequencing (RNA-Seq) was performed on mRNA samples extracted from the strain BY4741 AND YHJ7. Yeast cells were grown overnight in 25 ml YPD medium at 30 °C and 200 rpm, then inoculated in fresh medium (initial OD_600_ ∼ 0.05) for 18 h to early stationary phase (final OD_600_ ∼ 12). For each RNA-seq library, two biological replicates were taken and pooled for sequencing on an Illumina HiSeq2000. The raw sequencing data have been deposited in the National Center for Biotechnology Information Gene Expression Omnibus under accession number GSE54433. The genome sequence of *S. cerevisiae* strain S288c (sacCer3/SacCer_Apr2011) and its annotations were retrieved from Illumina’s igenome project (http://support.illumina.com/sequencing/sequencing_software/igenome.html, last accessed September 17, 2014). Both the processed reads of BY4741 and YHJ7 were mapped to the S288c genome using Tophat (V2.0.4). After that, gene expression levels were estimated as FPKM (fragments per kilobase of exon per million fragments mapped) values and differential expressed genes were identified by the Cufflinks (v2.0.2) package (Trapnell et al. 2012). For de novo transcriptome analysis, processed RNA-seq reads were assembled using the Trinity pipeline (Haas et al. 2013). The best protein-coding transcripts were identified and aligned back to the assembled genome using the pipeline included scripts. Enrichment of GO terms and KEGG pathways was performed in the GO terms finder in SGD website and DAVID functional annotation tool (Huang da et al. 2009).

### Comparative Genomics and Phylogenetic Analysis

The *S. cerevisiae* genomes were downloaded from GenBank (ftp.ncbi.nlm.nih.gov/genbank/genomes/Eukaryotes/fungi/Saccharomyces_cerevisiae, last accessed September 17, 2014) and SGD (www.yeastgenome.org/download-data/sequence, last accessed September 17, 2014). Multiple alignments of these genomes were determined using Mugsy (Angiuoli and Salzberg 2011) (aligned by NUCmer with default parameters). SNPs between any pair of these genomes were extracted and concatenated for phylogenetic analysis. To conduct comparative analysis of the SNP/InDels between S288c, K7, YHJ7, and YJSH1, the pairwise genome alignment of these four strains was aligned using NUCmer, then SNP/InDel was called using “show-snp” in the MUMmer package. Multiple genome alignment was generated to detect conserved genome sequences between YHJ7 and other strains (K7, YJSH1, and S288c), and only DNA regions larger than 200 bp were considered. Conserved genomic regions among all or some of the four strains were visualized using Circos 0.66 (http://circos.ca).

We used the 13 phylogenetic informative genes identified by Ramazzotti et al. (2012) to infer the phylogenetic relationships for YHJ7 and 15 other *S. cerevisiae* strains. The nucleotide sequences of the 13 genes from S288c were used as query sequences to search their homologous sequences in other strains. Their phylogenetic tree was reconstructed by using both Neighbor-Joining (NJ) method and Maximum Likelihood (ML) method. The evolutionary distances were computed using the Jukes–Cantor method. All positions containing gaps and missing data were eliminated. The NJ tree was built in MEGA5 with 1,000 bootstrap replicates (Tamura et al. 2011). ML tree was reconstructed using Phyml with 100 bootstrap replicates (Guindon and Gascuel 2003).

## Results and Discussions

### Genome Sequencing, Assembly, and Annotation

We sequenced the whole genome of a haploid *S. cerevisiae* strain YHJ7, isolated from Huangjiu fermentation, by Illumina method (see Materials and Methods). A total number of 18.3 million of 100-bp paired-end reads were generated, which represented more than 150-fold coverage of reference genome of the laboratory strain S288c (table 1). A hybrid assembly strategy by combining de novo and reference-guided assembly of reads was performed using Velvet and CLC genome workstation (see Materials and Methods). Our de novo assembly yielded a genome sequence of 11.5 Mb (1,012 contigs, *N*_50_ > 30 kb), which is very similar to the nuclear genome size of the reference strain S288c (12.1 Mb). All contigs of YHJ7 were then placed into corresponding position of 16 chromosomes of S288c.

**Table 1:** Summary of Sequencing Data

Library	Total Reads (Millions)	Mapped Reads (%)	Contigs (*N*^50^)
DNA-seq	18.3	97.77	1,012 (30,527)^a^
RNA-seq	10.4	96.86	9,395^b^

^a^Contigs were assembled by de novo methods, these contigs were placed into their chromosomes as described in Materials and Methods.

^b^Some genes have more than two transcript isoforms.

Genes in the genome of the YHJ7 strain were predicted by using a combination of ab initio and alignment based approaches (see Materials and Methods). Approximately, 5,500 genes were predicted in the YHJ7 strain (excluding those dubious ORFs in S288c). To provide further support for the gene annotation, we sequenced all cDNA for the YHJ7 strain growth on YPD medium with RNA-seq method. RNA-seq reads were assembled into transcripts using Trinity (Haas et al. 2013), and almost all RNA transcripts can be aligned to genome contigs. For those ORFs covered by mRNA transcripts, but were not presented in S288c, we manually checked and verified them by polymerase chain reaction and Sanger sequencing. Those ORFs were thus considered as strain-specific genes (supplementary table S1, Supplementary Material online).

### SNPs and InDels Identified between Chinese Rice Wine Strain and Other Strains

**Fig. 1.—:**
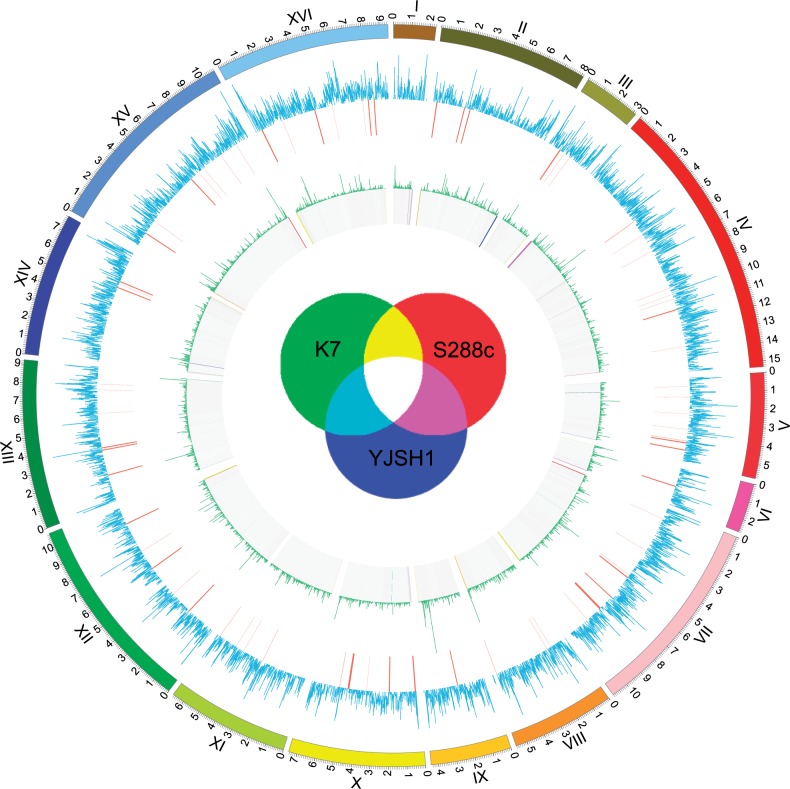
Genomic variations of YHJ7 comparing to S288c, K7, and YJSH1. Circle 1: The 16 chromosomes of YHJ7. Circle 2: Density of SNPs (blue) in YHJ7 compared with S288c. The SNP/InDel density was smoothed by a sliding window of the size 2 kb and step size 1 kb to reduce noise. Circle 3: Large deletions (>100 bp, red) in YHJ7 compared with S288c. Circle 4: Density of SNPs (green) in YHJ7 compared with K7. Circle 5: Conserved genomic regions (>200 bp) in YHJ7 compared with three other strains (S288c, K7, and YJSH1). Different conservation levels of genomic regions in YHJ7 are illustrated as different colors explained by the Venn diagram in the center. Specifically, if a genomic region in YHJ7 is only shared with S288c, it is shown in red. Similarly, K7—green; YJSH1—blue. The colors of overlapping circle in the center represent genomic regions in YHJ7 shared with two or three other strains, for example, a genomic region in YHJ7 is shown in purple if it is also found in S288c and YJSH1, and in white if it is conserved among all four strains.

To explore the genomic variations during the diversification of different *S. cerevisiae* strains, we identified SNPs and small InDels (≤100 bp) by pairwise comparing of YHJ7 with three other strains: The lab strain S288c, a Japanese sake strain K7, and a Chinese industrial bioethanol strain YJSH1. As shown in supplementary table S1, Supplementary Material online, we found that YHJ7 and K7 have the smallest number of SNPs (20,099), even though YHJ7 is geographically most close to YJSH1 (20,346 SNPs), suggesting that YHJ7 is genetically more closely related to K7 than to YJSH1. Comparing to S288c, the three Asian strains have similar numbers of SNPs and InDels (∼61,000 and ∼11,000, respectively; supplementary table S2, Supplementary Material online). In addition, approximately 82% of the SNPs (∼50,000) are common between any pair of two Asian strains, suggesting that the three Asian strains originated from a common ancestor that recently diverged from the ancestor of lab strain. Overall, the average SNP density between YHJ7 and S288c is 5.32 per kilobase throughout the genome. However, nonrandom distribution of SNPs and InDels was observed throughout the *S. cerevisiae* genome (fig. 1). About 50% of the total detected SNPs (30,099) between YHJ7 and S288c are found within the known protein-coding regions, and about 30% of them resulted in nonsynonymous substitutions. Similar to a previous observation on a Chinese industrial bioethanol strain YJSH1 (Zheng et al. 2012), the SNPs tended to accumulate within duplicated genes (such as *HXT*3, *PDR*5, and *FLO*5).

To detect large InDels (>100 bp), we used DELLY (Rausch et al. 2012) and Pindel (Ye et al. 2009) to identify structural variations from DNA sequencing paired-end reads. We detected 87 large InDels (up to 11.5 kb) between YHJ7 and S288c. Genes that have overlaps with these large InDels were listed in supplementary table S3, Supplementary Material online. Large InDels are highly enriched in subtelomeric regions, approximately 30-kb regions near the ends of chromosomes (fig. 1), such as the left arm of Chromosome II (5.9 kb), the right arm of Chromosome VII (11.8 kb), and the left arm of Chromosome XVI (6.2 kb). In addition, the comparison of genome sequences among YHJ7, K7, and YJSH1 also showed that large InDels are mainly found in subtelomeric regions (fig. 1). These results reinforced that the subtelomeric regions are a major source for divergence of genome sequences and gene content in *S. cerevisiae* and might have contributed to strain-specific adaptation process. Moreover, these large InDel regions mainly consist of repetitive elements. For example, the 11.5-kb deletion in Chromosome X contains four transposable element genes (supplementary fig. S2, Supplementary Material online). However, the functional effect of these large InDel regions on Huangjiu strain remains unknown.

To infer the potential functional impacts of the nonsynonymous SNPs and InDels identified in the YHJ7 strain, we have conducted GO term enrichment analysis. Our GO analysis revealed that the genes containing nonsynonymous SNPs and InDels are significantly enriched in molecular functions related to DNA binding, tansmembrane transporter activity, and oxidoreductase activity. It is worth noting that four genes (*GLN3*, *GAT1*, *GZF3*, and *DAL80*) involved in global regulation of nitrogen metabolism are highly enriched with nonsynonymous SNPs. *Saccharomyces cerevisiae* prefers to use nitrogen-rich sources, such as glutamine, asparagine, and ammonium. When these nitrogen-rich sources are unavailable, *S. cerevisiae* can use alternative nitrogen-poor sources, such as urea and arginine. Relief of nitrogen repression at the transcriptional level due to absence of nitrogen-rich sources is called nitrogen catabolite repression (NCR) (Hofman-Bang 1999). Mutations in the upstream regulators of NCR could significantly alter the amino acids profile of Huangjiu product, which plays important roles in formation of its flavor characteristics (Zhao et al. 2013).

We also examined the enrichment of nonsynonymous SNPs and InDels in pathways from KEGG database using the DAVID functional annotation tool. Our analysis showed that genes in six pathways (Meiosis, cell cycle, MAPK signaling pathway, nonhomologous end-joining, GPI-anchor biosynthesis, and regulation of autophagy) were enriched with nonsynonymous SNPs and InDels (*P* < 0.05). The high osmolarity glycerol (HOG) pathway has been suggested as the key MAPK signaling transduction pathway to sense and respond to hyperosmotic stress by regulating the transcription of multiple genes (O’Rourke et al. 2002). As Huangjiu was brewed with high concentration of sugar substrate (digested from rice starches) in fermentation mash, enrichment of nonsynonymous SNPs and InDels in the HOG pathway in YJH7 suggested a possible role of these genomic variations in its adaptive evolution under high osmotic conditions (Li et al. 2013).

### Evolution of Gene Content in the YHJ7 Strain

To study the evolution of gene content in different yeast strains, we identified the genes that are specifically present or absent in YHJ7 relative to S288c and K7. The two Asian strains have very similar gene contents. There is only one gene g1123 (chr4:779331–779774) that is present in YHJ7 but is absent in K7. The presence of g1123 in YHJ7 could be due to gene gain in YHJ7 or gene loss in K7. To elucidate the origin and evolution of g1123, we searched for homologous sequences from the genomes of other *S. cerevisiae* strains and other species, and we reconstructed their evolutionary history. The homologs of g1123 were found in many other *S. cerevisiae* strains, as well as a close relative species *Saccharomyces mikatae* (supplementary fig. S3, Supplementary Material online). As the homolog of g1123 is not identified in a third species, suggesting that g1123 is a novel gene in *Saccharomyces* species and has been lost in many strains, including K7. The function of g1123 has not been described and it would be interesting to learn its functional roles in future studies.

**Fig. 2.—:**
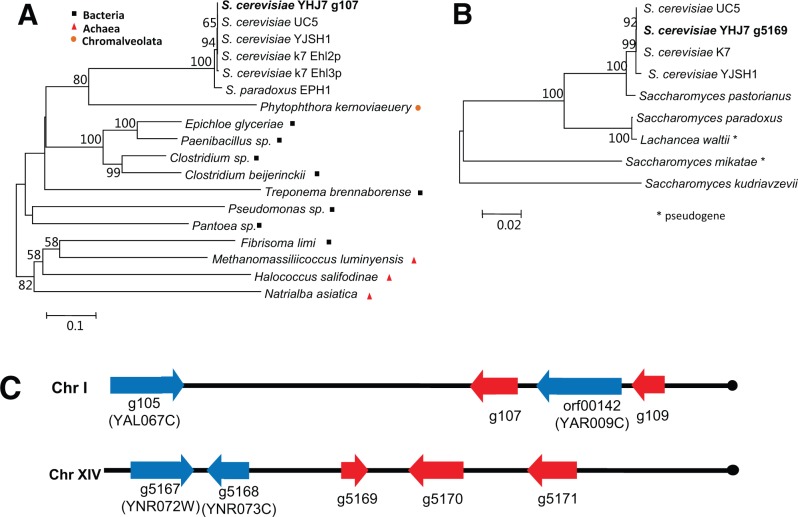
Origin and evolution of YHJ7 strain genes absent in S288c. (*A*) The YHJ7 g107 gene was likely originated from bacteria by horizontal gene transfer. (*B*) The homologous sequences of YHJ7 g5169 gene were found in several sake strains as well as other *Saccharomyces* sensu stricto species, but are absent in many *S. cerevisiae* strains. The lost of g5169 in S288c is probably due to the highly dynamic activity of subtelomeric region. The evolutionary history was inferred using the NJ method with 1,000 replicates of bootstrap test. The tree is drawn to scale. (*C*) YHJ7 strain-specific genes located in the subtelomeric regions of chromosomes. The arrows indicate the direction of transcriptions. Genes that are specific to YHJ7 relative to S288c were shaded in red. Genes with orthologous sequences in S288c were shaded in blue. The black dot indicates the end of a chromosome.

Comparison of gene content between YHJ7 and S288c leads to identification of nine genes that are present in YHJ7 but are absent in S288c, including the g1123 gene (supplementary table S1, Supplementary Material online). Seven of the nine genes were found to be actively transcribed according to our RNA-seq data. We conducted evolutionary analysis for all of these genes using the method similar for the study of the g1123 gene. Based on the presence/absence pattern of these genes and topology of phylogenetic trees, we found that the homologous sequences of all these YHJ7 genes are also present in several other *S. cerevisiae* strains, and at least one closely related species, such as *Saccharomyces paradoxus*, *Saccharomyces kudriavzevii*, and *S. mikatae* (fig. 2*A* and *B*). Therefore, it is reasonable to conclude that these genes have been present in the common ancestor of extant *S. cerevisiae* strains, but have been lost in the S288c strain during its evolutionary process.

We did not find homologous sequences of two YHJ7 genes (g107 and g1797) in almost all other eukaryotic species. However, the two genes share high sequence identities (>50%) with their bacterial counterparts (fig. 2*A*). Therefore, g107 and g1797 were likely originated from bacteria by horizontal gene transfer prior to divergence of *S. cerevisiae* strains. Furthermore, we found that five of the nine genes are located within the subtelomeric regions (fig. 2*C*). Subtelomeric regions have constantly experienced shortening and elongation processes, which is an important mechanism of telomere length maintenance (Lin and Li 2011). As a consequence, genes in subtelomeric regions have been undergoing frequent duplication and loss events (Lin and Li 2011). The homologous sequences of the five YHJ7 genes located in subtelomeric regions are present in several closely related strains of YHJ7, but are absent in many other *S. cerevisiae* strains. Previous studies indicated that gain or loss of genes located in subtelomeric regions in different *S. cerevisiae* strains could be due to the plastic and dynamic nature of subtelomeric regions (Kass-Eisler and Greider 2000; Argueso et al. 2009; Dunn et al. 2012), although the possibility of adaptive significance of these genes cannot be excluded.

To infer the possible functions of these YHJ7 genes that are absent in S288c, we searched for the annotated protein domains encoded by these genes from Pfam database or the documented functions of their homologous in other strains or species. Based on the domain search results by Pfam, the YHJ7 g107 gene encodes a protein that contains an Epoxide hydrolase N terminus domain, which might be involved in catalyzing the addition of water to oxirane compounds. The introgression between *S. paradoxus* and industrial *S. cerevisiae* strains, including the sake strain K7, has been observed for the epoxide hydrolase gene (Akao et al. 2011; Dunn et al. 2012), which may be crucial for the detoxification of harmful epoxide compounds in wine and fermenting mash. Therefore, the acquisition of g107 from bacteria might have facilitated the adaptation of YHJ7 to the wine fermentation environment.

The protein sequences of YJH7 g5169 share significant similarity to the members of GPR1/FUN34/yaaH family, which is likely an acetate transporter. The presence of acetate transporter might enhance the ability of YHJ7 to remove toxic intracellular acetic acid, and improve tolerance of acetic acid (Sousa et al. 1996; Sousa et al. 1998). Homologues of g5169 were found in several closely related yeast species, but is absent in almost all *S. cerevisiae* strains examined except for some sake and Asian strains (supplementary fig. S4, Supplementary Material online). The specific role of YHJ7 g5169 requires further investigation, but the unique presence of this gene in several closely related Asian strains suggests its potential role in adaptation of these strains to Asian wine fermentation environment.

The YHJ7 g5170 gene encodes a protein that contains a highly conserved Amidase domain, which catalyzes the hydrolysis of amide according to Pfam database. The homologs of YHJ7 g5170 were found in several sake strains and closely related species, including a bottom-fermenting yeast strain of *Saccharomyces pastorianus*, which is used to brew beer (supplementary fig. S5, Supplementary Material online). Function characterization of g5710 homologs in *S. pastorianus* (*AMI1*) showed that Ami1p may hydrolyze some amides related to amino acid and niacin metabolism in the cell, and it was suggested that *AMI1* is important for lager beer fermentation in bottom-fermenting yeast (Yoshida et al. 2007). Therefore, the maintenance of *AMI1* homologous gene (g5170) in the YHJ7 strain might play a similar role in amino acid and niacin metabolism, which is important for the Huangjiu fermentation by YHJ7. In summary, based on available functional information, these genes unique to YHJ7 might directly or indirectly facilitate the adaptation of YHJ7 to the industrial wine fermentation environments.

Identification of deletions in YHJ7 by next-generation sequencing methods allows us to analyze the genes that are present in S288c or K7, but are absent in YHJ7 (see Materials and Methods). We found one gene, YHR213W-B, which is present in K7 but is absent in YHJ7. This gene is located in a 3371-bp deletion, but its function is still unknown. We also found many transposable elements are present in K7 but are absent in YHJ7, for example: K7_YCLCTy4-1, K7_YDRCdelta15, K7_YELWdelta9, and K7_YOLCsigma1 (supplementary table S3, Supplementary Material online). However, we cannot exclude the possibility that the variations of repeat regions may be due to the false assembly of short reads of Next-Generation Sequencing technology. Compared with S288c, we identified 62 genes that are present in S288c but are absent in YHJ7 (supplementary table S3, Supplementary Material online). GO term analysis shows that these genes were enriched only at one biological process transposition (*P* < 1 e-10), a process involved in mediating the movement of DNA between nonhomologous sites. Thus, most of these genes are transposable element genes, for example, the largest deletion (11.5 kb) in Chromosome X contains four transposable element genes (supplementary fig. S2, Supplementary Material online). Similar to the many gene loss in subtelomeric regions in the S288c strain, the intrinsic plastic and dynamic nature of the subtelomeric regions account for many gene loss in YHJ7 strains. Interestingly, we found a member of the AAD (aryl-alcohol dehydrogenases) family gene, *AAD10*, is absent in the YHJ7 strain. In contrast, a *novel AAD* gene has been identified in the wine strain AWRI796 (Borneman et al. 2011). AAD enzymes were involved in converting aldehydes and ketones into their corresponding aromatic alcohols. Therefore, the presence or absence of these AAD genes in different industrial *S. cerevisiae* strains may have a direct impact on the profile of volatile aromas produced during wine fermentation, and leads to strain-specific aroma characteristics that are vitally important to wine sensory quality.

### Transcriptome Analysis of Chinese Rice Wine Strain Growing on Rich Medium YPD

To further examine the impacts of genome sequence divergence on the phenotypic evolution of YHJ7, we conducted a comparative transcriptome analysis between YHJ7 and a laboratory strain BY4741 (an S288c isogenic strain) under the same rich medium YPD. Overall, the global gene expression pattern between YHJ7 and BY4741 is highly similar (the Spearman correlation coefficient ρ = 0.325, *P* < 2.2 e-16). However, we found 39 genes are significantly differentially expressed between the two strains (supplementary table S4, Supplementary Material online). These genes fell into several interesting categories (ten without molecular functional annotation). Interestingly, several genes, such as *HXT2*, *HXT7*, and *HXT4*, are responsible for transporting hexoses (mainly glucose) across cellular membrane (table 2). Although the *S. cerevisiae* genome contains 18 genes encoding hexose transporter proteins (Hxt) (Lin and Li 2011), only six of them (Hxt1-4 p, Hxt6p, and Hxt7p) play major roles in transporting glucose across cellular membrane (Reifenberger et al. 1997). The glucose affinity of the six major transporters is quite different. Specifically, Hxt1p and Hxt3p are low-affinity carriers (K_m(glucose)_-values are 100 and 30–60 mM, respectively); Hxt2p and Hxt4p have an intermediate affinity (K_m_-value = 10 mM) and Hxt6p and Hxt7p are high-affinity carriers (K_m_-value is 1–2 mM) (Reifenberger et al. 1997). Based on studies of the lab strain S288c, it was found that the expression of these *HXT* genes is tightly controlled by the concentration of extracellular glucose (Ozcan and Johnston 1995). Genes encoding low affinity transporter (*HXT1* and *HXT3*) are highly expressed under high glucose concentrations, whereas *HXT6*, *HXT7*, *HXT2* and *HXT4* are repressed by high glucose concentrations but are induced by low glucose concentrations. Interestingly, four *HXT* genes (*HXT6*, *HXT7*, *HXT2*, and *HXT4*) have significant higher expression levels in the YHJ7 strain than that in S288c under growth in rich media. Considering that the copy number of *HXT* genes has a positive correlation with ethanol production efficiency (Lin and Li 2011), it is possible that all major *HXT* genes are actively transcribed in YHJ7 to obtain a faster glucose transportation and to achieve better fermentation efficiency. This observation was consistent with that the growth rate of YHJ7 was substantially higher than laboratory strains in the YPD media (data not shown). Therefore, the expression regulation of *HXT* genes by glucose in YHJ7 might have been modified to facilitate more efficient alcoholic fermentation.

**Table 2:** Function Enrichment Analysis of Differentially Expressed Genes in YHJ7 Compared with S288c

GO Term	No. of Genes (Total Genes)	*P* value^a^	Gene Names
Hexose transmembrane transporter activity	3/39	2.5e-3	*HXT*2, *HXT*4, *HXT*7
transporter activity	10/39	1.54e-3	*BIO*5, YOL163W, YOL162W, etc.
Oxidoreductase activity, acting on the CH–OH group of donors	5/39	2.2e-3	*AAD*10, *LEU*2, *BDH*2, *ADH*7, YJR096W

^a^The significantly differential expressed genes were identified at a false discovery rate <5%.

On the contrary, many genes involved in oxidoredutase activity, acting on CH–OH group of donors have been downregulated in YHJ7 (table 2), such as those genes of aryl-alcohol dehydrogenase (*AAD10*), alcohol dehydrogenase (*ADH7*), and 3-isopropylmalate dehydrogenase activity (*LEU2*). These genes might affect the brewing of Huangjiu by accumulating higher concentrations of high alcohol/aldehyde. The consumption of Huangjiu with high concentrations of these compounds might cause headache, which obstacles the popular consumption of Huangjiu.

### The Origin and Evolution of Chinese Rice Wine Strain YHJ7

**Fig. 3.—:**
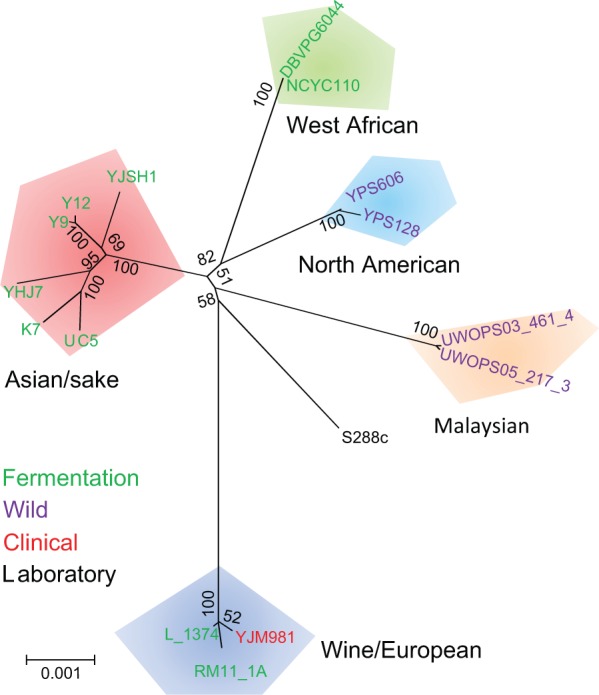
Phylogenetic tree of 16 representative strains of *S. cerevisiae* from various geological locations. The evolutionary history was inferred using the NJ method with 1,000 replicates of bootstrap test based on concatenated nucleotide sequences of 13 phylogenetic informative loci. The tree was drawn to scale. Except for the lab strain S288c, the 15 other *S. cerevisiae* strains form five well-supported clades. The Huangjiu strain YHJ7 is grouped within the clade of Asian sake strains and is mostly closely related the sake K7 and UC5 strains.

To determine the evolutionary origin of YHJ7, we reconstructed a phylogenetic tree of YHJ7 with 15 other representative strains of *S. cerevisiae* from five well-defined geographically isolated lineages (Liti et al. 2009). Given the mosaic nature of genomes of many *S. cerevisiae* strains (Liti et al. 2009), we inferred the phylogeny of the 16 strains using the nucleotide sequences of 13 phylogenetic informative genes, which were suggested as the most reliable molecular markers to infer the genetic relationships of *S. cerevisiae* strains (Ramazzotti et al. 2012). We reconstructed phylogenetic trees of the 16 strains based on concatenated sequence alignment of the 13 genes using both NJ and ML methods (fig. 3). The two methods yielded identical tree topologies supporting the reliability of phylogenetic reconstruction. The strains from five different geographical locations (Asian sake, West African, North American, Malaysian, and Wine/European) form five well-supported clades, which is consistent with previous studies based on genome-wide segregation sites (Ramazzotti et al. 2012). The YHJ7 is found in a well-supported clade that includes Japanese sake strains K7, UC5, a Chinese bioethanol strain YJSH1 (Zheng et al. 2012), an Indonesian yeast cake strain Y9, and a palm wine strain Y12 from Ivory Coast, Africa (Liti et al. 2009). Among these strains, YHJ7 is most closely related to the Japanese sake strains K7 and UC5, suggesting that these three strains shared a most recent common ancestor. In addition, the phylogenetic tree based on whole-genome’s SNP data further confirmed the above observations (supplementary fig. S6, Supplementary Material online). Considering that Huangjiu has been brewed and consumed in China for more than 5,000 years (McGovern et al. 2004) and the Japanese learned rice cropping and brewing from the Chinese and/or the Koreans (Teramoto et al. 1993), these results indicating that the Japanese sake strains may be derived from Chinese Huangjiu strains.

Fay and Benavides identified two domestication events of *S. cerevisiae* strains: One for the sake strains and the other for wine strains based on genetic survey of 5 loci in 81 strains of *S. cerevisiae*, (Fay and Benavides 2005). Our study revealed that the Huangjiu YHJ7 strains and Japanese sake strains shared a most recent common ancestor, suggesting that YHJ7 and sake strains were originated from a same domestication event. The average number of differences per synonymous site between YHJ7 and the two sake strains K7 and UC5 is 4.93 × 10^−^^3^ based on the 13 phylogenetic informative genes. It has been estimated that the point mutation rate in *S. cerevisiae* is 1.84 × 10^−^^10^ per base pair per generation (Fay and Benavides 2005). Considering that the generation time of *S. cerevisiae* is 90 min, which is equivalent to 16 generations per day. Therefore, we estimated that the divergence time between YHJ7 and the common ancestor of Japanese sake strains is approximately 2,300 years. Because the actual generation number per year probably might be less than theoretical estimation, the divergence time between YHJ7 and Japanese sake strains obtained in this study only provided a minimum estimation. The earliest written record about the first brewing of sake in Japan could be traced back to 2,500 years ago when wet rice cultivation became prevalent according to Japan Sake and Shochu Makers Association (http://www.japansake.or.jp/, last accessed September 17, 2014). Therefore, our evolutionary study supports this scenario, which is the first molecular clock evidence to unravel that sake strains were derived from Chinese Huangjiu strains and were introduced to Japan at least 2,300 years ago.

## Conclusions

During Huangjiu fermentation, the YHJ7 cells have been subjected to different kinds of stresses, especially the osmotic stress which is imposed by the high sugar content of must and ethanol produced during fermentation (Querol et al. 2003). Therefore, to adapt to its special niches, the genome of YHJ7 has been shaped by strong selective pressures under wine fermentation environment. In addition, YHJ7 also evolved some strain-specific traits including production of Huangjiu-specific sensory characteristics and nutrients, as well as undesired byproducts such as higher alcohols and ethyl carbamate. Through comparative studies of genomes and transcriptomes between YHJ7 and other strains, we identified many genomic and transcriptional variations that might be directly or indirectly related to the adaptation of YHJ7 to Huangjiu fermentation environments and evolution of strain-specific straits, such as the improved sugar uptake, adaptation to high osmolarity environment, detoxification of harmful epoxide compounds, and removal of toxic intracellular acetic acid. Although future studies are still needed to further examine the functional effects of these genetic variations, our study has shed lights on the evolution of yeast genome under wine fermentation environments and provided a valuable resource for comparative genomics study and genetic manipulation to improve the Chinese Huangjiu quality.

## Supplementary Material

Supplementary data set S1, figures S1–S6, and tables S1–S4 are available at *Genome Biology and Evolution* online (http://www.gbe.oxfordjournals.org/).

Supplementary Data
