# Supplementary material for: Genomic Evolution of Saccharomyces cerevisiae under Chinese Rice Wine Fermentation
Source: Genome Biol Evol. 2014 Sep 10;6(9):2516–26. doi: 10.1093/gbe/evu201 (PMC4202337; doi:10.1093/gbe/evu201)
Supplement: Supplementary Data [file supp_6_9_2516__index.html]

Genomic Evolution of Saccharomyces cerevisiae under Chinese Rice Wine Fermentation — Supplementary Data 

# Genomic Evolution of *Saccharomyces cerevisiae* under Chinese Rice Wine Fermentation

## Supplementary Data

files

**Files in this Data Supplement:**

- Supplementary Data - xls file
- Supplementary Data - doc file
