# Supplementary material for: Genomic Evolution of Saccharomyces cerevisiae under Chinese Rice Wine Fermentation
Source: Genome Biol Evol. 2014 Sep 10;6(9):2516–26. doi: 10.1093/gbe/evu201 (PMC4202337; doi:10.1093/gbe/evu201)
Supplement: Supplementary Data [file supp_evu201_Supplementary_materials_20140722.doc]

**Supplementary materials**

**Genomic Evolution of *Saccharomyces cerevisiae* under Chinese Rice Wine Fermentation**

Yudong Li^1*^, Weiping Zhang^2^, Daoqiong Zheng^3^, Zhan Zhou^3^, Wenwen Yu^1^, Lei Zhang^1^, Lifang Feng^1^, Xinle Liang^1^, Wenjun Guan^3^, Jingwen Zhou^2^, Jian Chen^2*^, Zhenguo Lin^4, 5 *^

^1^ Department of Bioengineering, School of Food Sciences and Biotechnology, Zhejiang Gongshang University, Hangzhou, China 310035

^2^ Key Laboratory of Industrial Biotechnology, School of Biotechnology, Jiangnan University, Wuxi, China 214122

^3^ College of Life Sciences, Zhejiang University, Hangzhou, China 310058

^4^ Department of Biology, Saint Louis University, St. Louis, MO, USA 63103

^5^ Department of Ecology and Evolutionary Biology, Rice University, Houston, TX, USA 77005

*Corresponding authors:

Y. Li: Tel.: 86-571-88206636; Fax: 86-571-88206627; E-mail: [lyd@zjsu.edu.cn](mailto:lyd@zjsu.edu.cn);

J. Chen: Tel.: 86-510-85918312; Fax: 86-510-85918309; E-mail: [jchen@jiangnan.edu.cn](mailto:jchen@jiangnan.edu.cn);

Z. Lin: Tel.: 1-314-977-3909; Fax: 1-314-977-3658; E-mail: [zhenguolin@slu.edu](../../../../Downloads/zlin@rice.edu);

**Table S1 List of novel ORFs found in YHJ7 relative to S288c**

| **ORFs** | **Location** | **Pfam domain** | **Possible functions** |
| --- | --- | --- | --- |
| **g107** | chr1:204446-205579 | Epoxide hydrolase N terminus (EHN) domain | detoxification of harmful epoxide compounds in wine and fermenting mash |
| **g109** | chr1:208366-209154 | no match | hypothetical protein |
| **g1123** | chr4:779331-779774 | no match | hypothetical protein |
| **g1797 ^a^** | chr6:1416-1796 | no match | hypothetical protein |
| **g4507** | chr13:452022-452285 | no match | hypothetical protein |
| **g5169 ^a^** | chr14:757576-758403 | GPR1/FUN34/yaaH | acetate transporter |
| **g5170** | chr14:759784-761580 | Amidase | catalyzes the hydrolysis of an amide |
| ***g5171*** | chr14:763665-765350 | Asp | cell wall growth and maintenance |
| **g5682** | chr15:951442-952401 | no match |  |

^a^This ORF without support of RNA-seq reads

**Table S2**  Pairwise identification of SNPs/InDels among YHJ7, K7, YJSH1 and S288c.

|  | **YHJ7** | **K7** | **YJSH1** |
| --- | --- | --- | --- |
| **K7** | 20999/4951 |  |  |
| **YJSH1** | 20346/4916 | 23425/5793 |  |
| **S288c** | 61282/11024 | 61501/11299 | 61073/11258 |

**Table S3 List of deleted regions in YHJ7 relative to S288c or K7**

|  | **Deletion** | **Size** | **Location** | **Genes ^a^** |
| --- | --- | --- | --- | --- |
| S288c | Deletion_00001001 | 6268 | chrV:443364-449628 | YER138W-A |
|  | Deletion_00001273 | 373 | chrVII:518071-518432 | YGR014W |
|  | Deletion_00001557 | 943 | chrVIII:526248-526919 | YHR211W |
|  | Deletion_00000806 | 5414 | chrIX:205227-210641 | YIL082W |
|  | Deletion_00001570 | 7518 | chrX:21596-29114 | YJL214W/ YJL217W/ YJL216C/ YJL218W/ YJL215C |
|  | Deletion_00001681 | 11502 | chrX:472467-483969 | YJR030C |
|  | Deletion_aad10 | 1000 | chrX:727405-728271 | YJR155W (AAD10) |
|  | Deletion_00001923 | 792 | chrXI:647309-647946 | YKR102W |
|  | Deletion_00002305 | 5908 | chrXIII:357012-362918 | YMR046W-A |
|  | Deletion_00002369 | 201 | chrXIII:609473-609674 | YMR173W-A/ YMR173W |
|  | Deletion_00003068 | 223 | chrXVI:560582-560805 | YPR002C-A |
| K7 | K7_Deletion_1 | 214 | chrI:10396-10610 | K7_00014 |
|  | K7_Deletion_2 | 237 | chrI:19578-19815 | YAL061W |
|  | K7_Deletion_3 | 374 | chrII:779528-779902 | MAL33b |
|  | K7_Deletion_4 | 152 | chrIII:94551-94703 | YCLCTy4-1 |
|  | K7_Deletion_5 | 1048 | chrIV:939605-940653 | YDRCdelta15/YDRWdelta12/… |
|  | K7_Deletion_6 | 126 | chrIX:30168-30294 | K7_03399 |
|  | K7_Deletion_7 | 426 | chrV:149482-149908 | YELWTyl1-1/YELWdelta9 |
|  | K7_Deletion_8 | 582 | chrVI:4613-5195 | HXT9-2 |
|  | K7_Deletion_9 | 3371 | chrVIII:525530-528901 | K7_03371/ **YHR213W** /FLO5 |
|  | K7_Deletion_10 | 394 | chrX:1342-1736 | FSP2-1b |
|  | K7_Deletion_11 | 545 | chrX:708046-708591 | **SNO2**-2 |
|  | K7_Deletion_12 | 224 | chrXI:663550-663326 | K7_04475 |
|  | K7_Deletion_13 | 914 | chrXII:365072-365986 | YLRWsigma3/YLRWTy3 |
|  | K7_Deletion_14 | 131 | chrXIII:168013-168144 | YMLWTy1-1 |
|  | K7_Deletion_15 | 126 | chrXIII:746642-746516 | YHM2 |
|  | K7_Deletion_16 | 520 | chrXIV:691616-691096 | AGA1 |
|  | K7_Deletion_17 | 133 | chrXV:4770-4903 | HXT11-1a |
|  | K7_Deletion_18 | 115 | chrXV:92452-92567 | YOLCsigma1 |

^a^ The genes are overlapped with the deletions, and transposable element genes were not included.

**Table S4 Significant different expressed genes between rice wine and laboratory strains ^a^**

| **Systematic name** | **Gene name** | **Expression levels (log2 fold)** | ***P*-value**  **(q value)** | **Function** |
| --- | --- | --- | --- | --- |
| YAL061W | BDH2 | 792.96/48.85 (-4.02) | 1.00E-04  (0.027) | Putative medium-chain alcohol dehydrogenase |
| YAR031W | PRM9 | 6.65/0 | 1.00E-04  (0.027) | Pheromone-Regulated Membrane protein |
| YAR033W | MST28 | 9.49/0 | 5.00E-05  (0.012) | Putative integral membrane protein |
| YCL018W | LEU2 | 0/78.5 | 5.00E-05  (0.012) | Beta-isopropylmalate dehydrogenase (IMDH) |
| YCL069W | VBA3 | 0/4.64 | 2.00E-04  (0.036) | Vacuolar Basic Amino acid transporter |
| YCR105W | - | 21.92/0 | 5.00E-05  (0.012) | Alcohol DeHydrogenase |
| YCR106W | RDS1 | 11.35/0 | 5.00E-05  (0.012) | Putative zinc cluster transcription factor |
| YDR342C | HXT7 | 21.9/506.36 (4.53) | 1.00E-04  (0.021) | Hexose Transporter |
| YEL021W | URA3 | 0/158.46 | 5.00E-05  (0.012) | Orotidine-5'-phosphate (OMP) decarboxylase |
| YGL051W | MST27 | 18.75/0 | 1.00E-04  (0.021) | Putative integral membrane protein |
| YGL053W | PRM8 | 177.16/0 | 5.00E-05  (0.012) | Pheromone-regulated protein |
| YGR044C | RME1 | 410.63/4.59 (-6.48) | 3.00E-04  (0.049) | Zinc finger protein involved in control of meiosis |
| YGR065C | VHT1 | 49.1/1311.87  (4.74) | 0.00015  (0.029) | Vitamin H Transporter |
| YGR154C | GTO1 | 30.37/563.37 (4.21) | 5.00E-05  (0.012) | Omega-class glutathione transferase |
| YHR092C | HXT4 | 5.2/2928.57 (9.13) | 5.00E-05  (0.012) | High-affinity glucose transporter |
| YIL162W | SUC2 | 65.69/3651.76 (5.8) | 5.00E-05  (0.012) | sucrose hydrolyzing enzyme |
| YJR096W | - | 420.93/32.53 (-3.69) | 0.00025  (0.043) | putative xylose and arabinose reductase |
| YJR155W | AAD10 | 183.14/0 | 5.00E-05  (0.012) | aryl-alcohol dehydrogenase |
| YLR303W | MET17 | 0/30.11 | 5.00E-05  (0.012) | Methionine and cysteine synthase |
| YLR411W | CTR3 | 12.49/823.69 (6.04) | 5.00E-05  (0.012) | copper transporter |
| YMR011W | HXT2 | 66.22/6059.59 (6.52) | 0.00015  (0.029) | glucose transporter |
| YMR095C | SNO1 | 21.24/804.17 (5.24) | 5.00E-05  (0.012) | Protein of unconfirmed function |
| YNR056C | BIO5 | 9.19/1048.8 (6.84) | 5.00E-05  (0.012) | transmembrane protein involved in the biotin biosynthesis pathway |
| YNR058W | BIO3 | 25/398.48  (3.99) | 1.00E-04  (0.021) | 7,8-diamino-pelargonic acid aminotransferase |
| YNR074C | AIF1 | 30.34/0 | 5.00E-05  (0.012) | Apoptosis-Inducing Factor |
| YOL155C | HPF1 | 176.53/5686.49 (5.01) | 5.00E-05  (0.012) | Haze Protective Factor |
| YOL164W | BDS1 | 34.47/0 | 5.00E-05  (0.012) | Bacterially-derived sulfatase |
| YOR391C | HSP33 | 9.34/0 | 5.00E-05  (0.012) | Heat-Shock Protein |
| YPR194C | OPT2 | 14.6/291.63 (4.32) | 3.00E-04  (0.049) | Oligopeptide transporter |

^a^Those genes without molecular functional annotation or dubious genes were not listed.


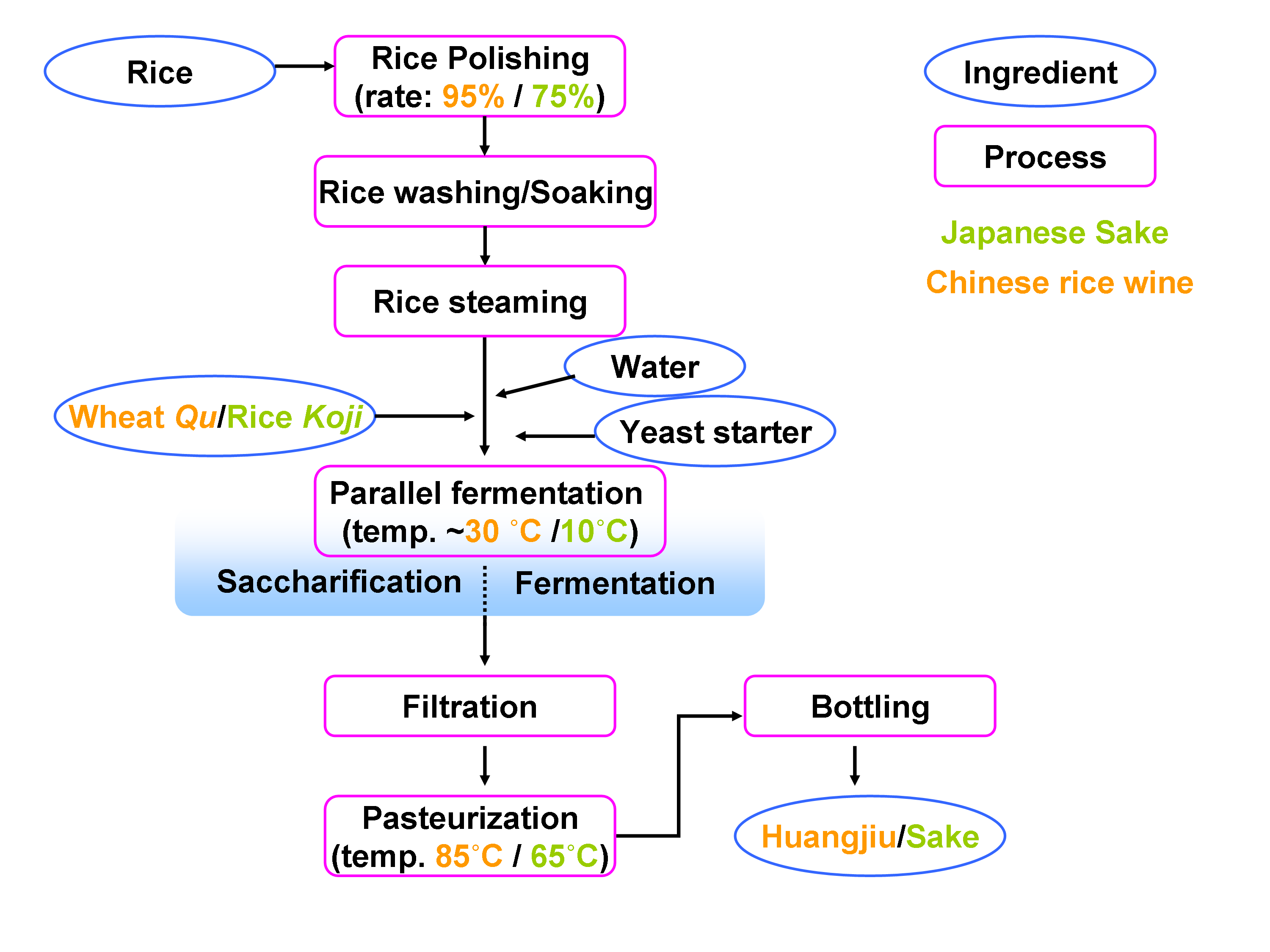


**Figure S1** **An Overview of the Chinese rice wine brewing process.** The differences between Sake and Huangjiu were indicated.


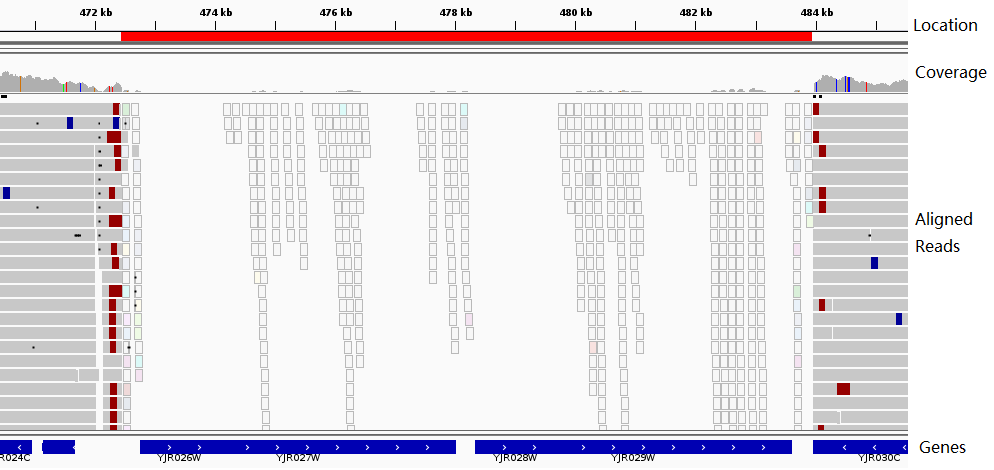


**Figure S2 The 11.5kb deletion on chromosome X in strain YHJ7 displayed in the integrative genomics viewer.** Aligned reads are displayed as gray bars; Red-colored reads indicate possible deletion. The deleted region is highlighted by red colour, and its read coverage is greatly lower than nearby regions.

**Figure S3 Phylogenetic tree of YHJ7 g1123.** The evolutionary history was inferred using the Neighbor-Joining method tree using the nucleotide sequence of YHJ7 g1123 and its homologous sequences. Evolutionary analyses were conducted in MEGA5.

**Figure S4 Phylogenetic tree of YHJ7 g5169.** The evolutionary history was inferred using the Neighbor-Joining method tree using the nucleotide sequence of YHJ7 g5169 and its homologous sequences. Evolutionary analyses were conducted in MEGA5.

**Figure S5 Phylogenetic tree of YHJ7 g5170.** The evolutionary history was inferred using the Neighbor-Joining method tree using the nucleotide sequence of YHJ7 g5710 and its homologous sequences. Evolutionary analyses were conducted in MEGA5.


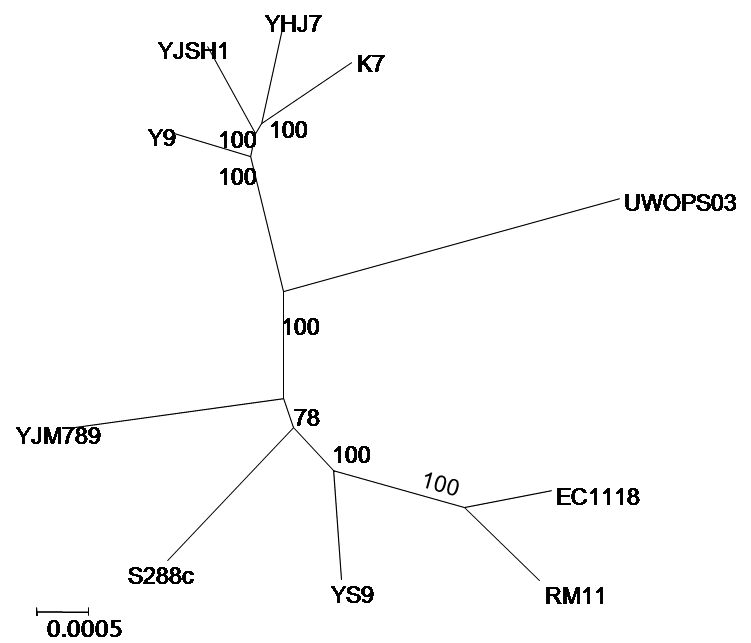


**Figure S6 Phylogenetic tree of different *S. cerevisiae* strains.** The tree was constructed by NJ methods based on the SNP data of the 11 strains’ whole genome sequences. There were a total of 161,482 positions in the final dataset.
